# Chemical proteomics enhances the understanding of 2AA stress in *Salmonella enterica*

**DOI:** 10.1128/msystems.00540-25

**Published:** 2025-05-29

**Authors:** Dominik Schum, Michaela K. Fiedler, Wangchen Shen, Stephan A. Sieber, Diana M. Downs

**Affiliations:** 1 School of Natural Sciences, Department of Bioscience, Chair of Organic Chemistry II, Center for Functional Protein Assemblies (CPA), Technical University of Munich (TUM) 686517, Garching, Bavaria, Germany; 2 Department of Microbiology, University of Georgia 189270, Athens, Georgia, USA; Academia Sinica Agricultural Biotechnology Research Center, Tainan City, Taiwan

**Keywords:** pyridoxal-phosphate-dependent enzymes, PLP, chemical proteomics, 2-aminoacrylate, RidA

## Abstract

Pyridoxal 5′-phosphate (PLP) is an essential cofactor for enzymes that catalyze diverse reactions in central metabolism. 2-Aminoacrylate (2AA) is a reactive enamine and an obligate catalytic intermediate in some PLP-mediated reactions. In the absence of the enamine/imine deaminase RidA, *Salmonella enterica* accumulates 2AA, which causes cellular stress. 2AA can attack PLP in the active site of some enzymes and covalently inactivate them by forming a 2AA-PLP adduct, which has already been characterized for some target enzymes *in vivo* and *in vitro*. The mechanism of 2AA attack suggests that a majority of cellular PLP-DEs would be targets of 2AA damage. Herein, a chemical proteomics workflow that uses PL (pyridoxal) probes to enrich PLP-DEs with a click chemistry-based protocol was implemented to investigate the global scale of 2AA damage in *S. enterica*. The results showed that PLP-DEs could be enriched in *S. enterica* with two different PL probes. When cells were labeled by providing a PL probe as the sole source of vitamin B6, several proteins were found to be more enriched when grown in conditions of high 2AA versus low 2AA stress. These data identified proteins that were previously shown to be attacked by 2AA as well as new candidate targets, demonstrating the usefulness of this approach to define the 2AA stress response with a global perspective. Growth analyses indicated that 2AA stress impacts the salvage of PL probes, suggesting that these and other PL probes will be valuable in future physiological studies to understand PLP salvage, a critical pathway in all organisms. In total, this study expands our understanding of 2AA metabolism and takes an initial step toward characterizing the global impact of 2AA stress in *S. enterica*.

**IMPORTANCE:**

Loss of RidA homologs results in 2-aminoacrylate stress in *Salmonella* and other bacteria. The stress is derived from the reaction of 2AA with a pyridoxal phosphate cofactor in metabolic enzymes, which inactivates the respective enzymes. This study uses a chemical proteomic method and, with an initial test case, explores the damage that is generated by 2AA on a global proteomic scale. This work provides a basis for probing the extent of 2AA stress in different organisms and for identifying the enzymes targeted by 2AA.

## INTRODUCTION

Microbial metabolism is a complex system of interwoven pathways coordinated by an intricate, multilayered network of regulation. The coordinated regulation of transcription, translation, and enzymatic activity in response to various cues generates a metabolic network that is responsive to changing environmental conditions and stresses. Many of the regulatory systems and activities that contribute flexibility to the network have been characterized over decades, and new paradigms continue to emerge. Despite the global level of metabolic responses, in general, each functional paradigm has been established and the mechanism defined by *in vitro* and *in vivo* approaches that focus on one or a few components of the system at a time (1, 2). Global approaches are often employed to efficiently define the breadth of the relevant phenomenon in the cell and expand the understanding of its contribution to cell fitness. The field of post-translational modifications illustrates this progression well, highlighting our understanding of protein phosphorylation, methylation, and acetylation at the mechanistic level in the context of single proteins (3). Once the relevant signature is defined (i.e., acetyl lysine group [4]), the prevalence, timing, and regulation of these modifications can be assessed with various proteomic approaches (5). The global data sets that result from these multiple studies provide a context on which models and testable hypotheses are built to explain the benefit of regulatory changes in optimizing the fitness of the organism.

**Fig 1:**
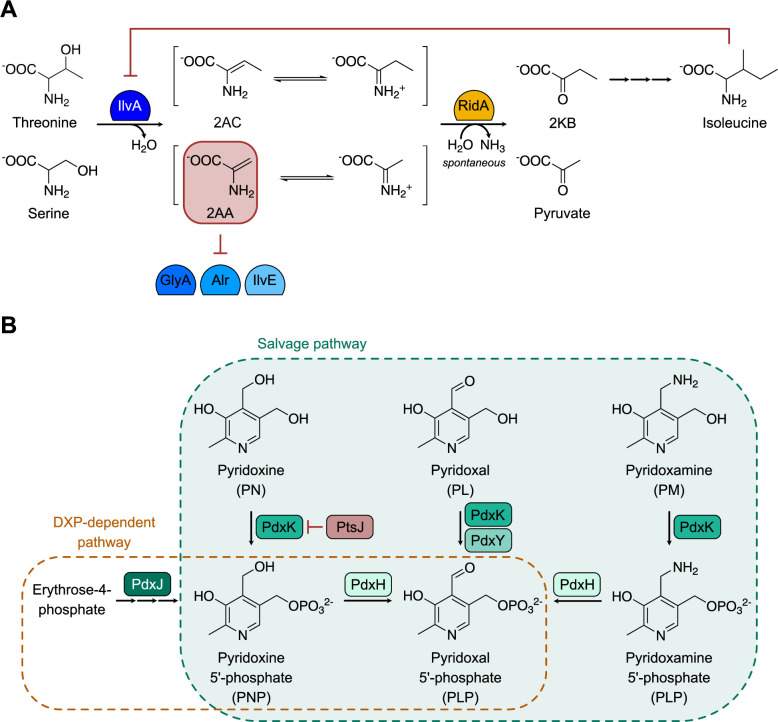
Source and consequence of 2AA stress in *Salmonella enterica*. (A) The RidA paradigm of 2AA stress in *Salmonella enterica* is schematically shown. Dome symbols represent the designated enzymes. IlvA is a dehydratase that uses threonine as a substrate in the biosynthesis of isoleucine. IlvA dehydrates threonine, or the alternative substrate serine, to generate the associated enamines (2-aminocrotonate or 2-aminoacrylate, respectively), which tautomerize to the respective imines. These short-lived intermediates are deaminated by solvent water or the RidA deaminase to generate the respective, stable ketoacids. In the absence of RidA, the enamines are accumulated. If free, 2AA can attack some PLP-dependent enzymes and covalently inactivate them as described in the text. Isoleucine allosterically inhibits IlvA, preventing the formation of both 2AC and 2AA. (**B**) Relevant components of vitamin B6 synthesis and salvage are schematically shown. Green shaded boxes depict enzymes in salvage (PdxK, PdxY, and PdxH) and synthesis by the DXP-dependent pathway present in *S. enterica* (PdxJ and PdxH). The red box shows PtsJ, a transcriptional repressor of PdxK. AC = aminocrotonate, 2AA = 2-aminoacrylate, and 2KB = 2-ketobutyrate.

While changes in the pattern of cellular components and/or metabolites can be the result of regulation, changes are also caused by stresses that perturb the metabolic network. Understanding of the 2-aminoacrylate stress paradigm has evolved in the past several years primarily from studies in *Salmonella enterica* that were initiated to define the function of the conserved RidA protein (6–8). RidA is the archetypical member of the Rid superfamily and is conserved in all domains of life (9, 10). In *S. enterica,* 2AA is derived primarily from the dehydration of serine by the serine/threonine dehydratase (IlvA, EC 4.3.1.19) (8). Serine is a secondary substrate of IlvA, which predominately dehydrates threonine to aminocrotonate (AC) in the first step of the biosynthetic pathway of the branched-chain amino acids, including isoleucine. The products of IlvA catalysis, AC and 2AA, spontaneously deaminate to their respective ketoacids (Fig. 1). The RidA protein enhances the rate of deamination and thereby prevents the accumulation of 2AA *in vivo* (11). Despite an estimated half-life of less than 3 seconds in aqueous solutions (12), there is sufficient 2AA accumulation *in vivo* that can cause damage in the absence of RidA. Deleterious effects of 2AA accumulation are found in multiple organisms and are best understood in *S. enterica* (Fig. 1A) (10, 13–15). IlvA catalyzes the formation of 2AA from endogenous (or exogenous) serine unless it is allosterically inhibited by isoleucine. In the absence of RidA, the reactive molecule accumulates and targets pyridoxal-phosphate-dependent enzymes (PLP-DEs). 2AA damages some PLP-DEs by a covalent modification that involves the formation of a 2AA-PLP adduct, which inactivates the enzyme. Such an attack reduces the population of the active protein and in some cases results in a detectable growth phenotype due to decreased enzymatic activity. This mechanism has been demonstrated to occur both *in vitro* and *in vivo* for the enzymes IlvE (branched- hain amino acid aminotransferase, EC 2.6.1.27), GlyA (serine hydroxymethyltransferase, EC 4.3.1.18), IscS (cysteine desulfurase, EC 2.8.1.7), Alr (alanine racemase 1, EC 5.1.1.10), and AspC (aspartate aminotransferase, EC 2.6.1.1) from *S. enterica* (16–19). Other PLP-DEs appeared to be immune to the damage, including YggS (pyridoxal phosphate homeostasis protein) and GabT (4-aminobutyrate aminotransferase, EC 2.6.1.19) (Shen and Downs, unpublished). However, the accumulation of the reactive metabolite 2AA has the potential to alter the global proteome.

The direct target of 2AA damage in the cell is PLP, the biologically active form of vitamin B6, which is a collective term for six vitamers: pyridoxal (PL), pyridoxine (PN), pyridoxamine (PM), and their phosphorylated derivatives (PLP, PNP, and PMP). Cells can obtain PLP by *de novo* synthesis and/or salvage of one or more vitamers from the environment (Fig. 1B). Enzymes that have been assigned a role in vitamin B6 salvage in *S. enterica* include PdxK (PN/PL/PM kinase, EC 2.7.1.35) (20), PdxY (PL kinase, EC 2.7.1.35) (21), and PdxH (PNP/PMP oxidase, EC 1.4.3.5) (22, 23). The latter enzyme catalyzes the final step in PLP biosynthesis in some organisms including *S. enterica* and *Escherichia coli*. Despite the critical nature of PLP, significant gaps remain in our knowledge about the synthesis, salvage, and homeostasis of vitamin B6 and the regulation of these processes.

The mechanism of 2AA-dependent inactivation of PLP-DEs was defined *in vitro,* and damage to several individual enzymes has been characterized in *S. enterica* and other organisms (7, 15, 16, 18, 24). Since many questions that remain about 2AA metabolic stress are too laborious to answer with a “one-enzyme-at-a-time” approach, a new approach is needed to generate a global snapshot of the effect of 2AA stress across organisms. Activity-based protein profiling (ABPP) with PL probes (25) has the potential to provide the means to assess the 2AA-damaged proteome. This study was initiated to determine the impact of 2AA stress on the proteome of *S. enterica*. Specifically, we sought to define a workflow that would differentiate between PLP-DEs that have been damaged by 2AA and those that have not been damaged. In this effort, we took advantage of the well-characterized 2AA stress paradigm of a *ridA* mutant of *S. enterica* and the current knowledge of PLP biosynthesis and salvage. The results herein contribute new information relevant to each of these areas.

## RESULTS AND DISCUSSION

### Growth analyses inform experimental design for PL labeling

**Fig 2:**
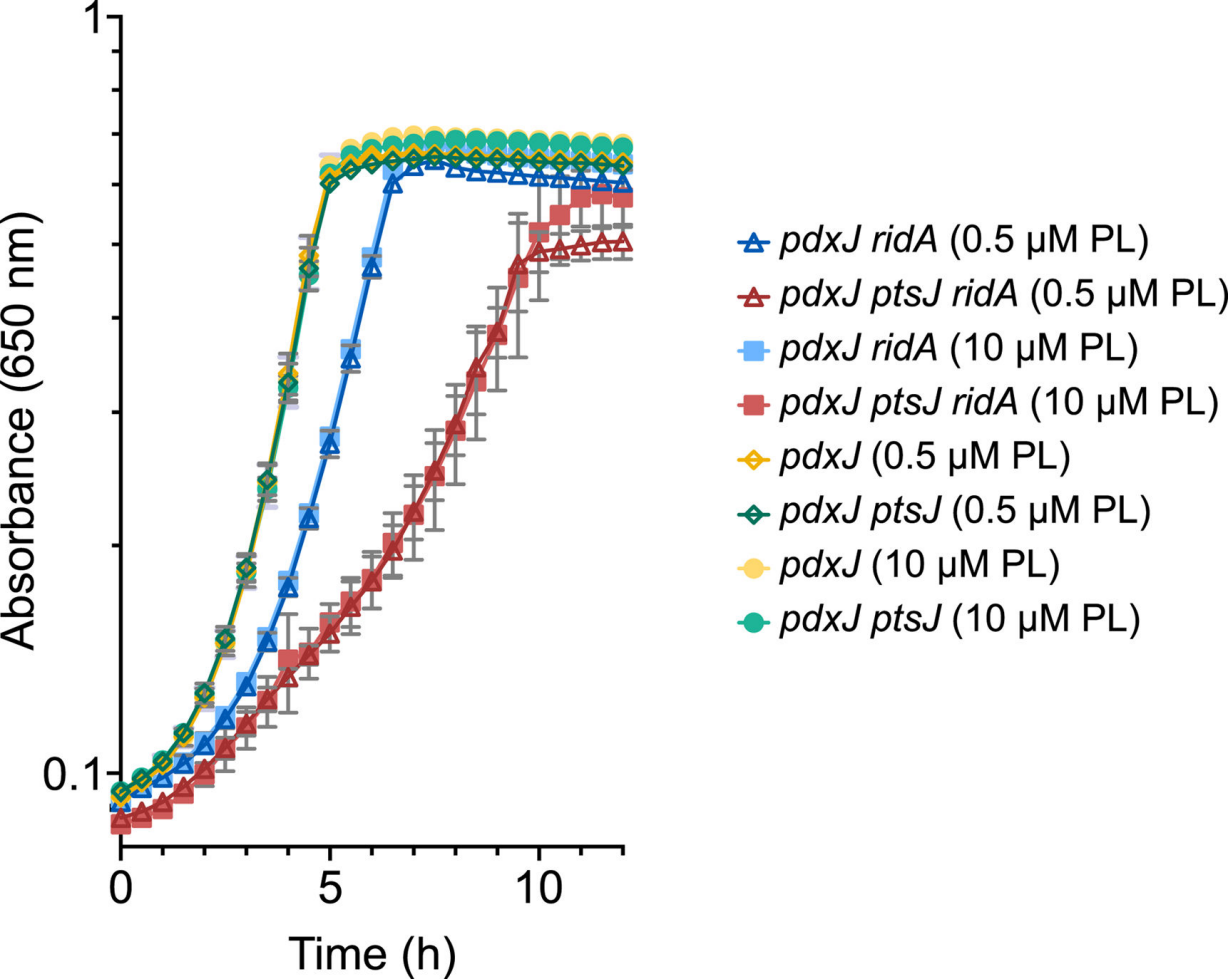
Loss of PtsJ does not facilitate PL salvage. Four mutant strains that require exogenous B6 (*pdxJ*, *pdxJ ptsJ*, *pdxJ ridA,* and *pdxJ ptsJ ridA*) were grown in minimal glucose (11 mM) medium with the addition of either 0.5 or 10 µM PL, as indicated. Growth was determined by monitoring the optical density at 650 nm. Strains are designated by the symbols in the included legend. Error bars represent standard deviation between *n* = 3 independent biological replicates. PL = pyridoxal.

Several analogs of vitamin B6 that can be used as PL probes in chemical proteomics/click chemistry protocols have been characterized. Two previously described PL probes, PL1 and PL2 (25), were chosen to label *S. enterica* strains and enrich for PLP-DEs under different conditions. Two mutant strains appropriate for this study (*pdxJ* and *pdxJ ridA*) were constructed, and growth studies were performed to optimize the labeling approaches. Each of the strains lacked PdxJ (pyridoxine 5′-synthase, EC 2.6.99.2), which prevented *de novo* synthesis of PLP. Additionally, one strain lacked RidA, resulting in the accumulation of varying levels of 2AA depending on the growth conditions. PL1 and PL2 were investigated for the ability to satisfy the vitamin B6 requirement of these two strains. As anticipated from studies on *E. coli* (25), PL1, but not PL2, allowed growth of both strains when provided as the sole source of vitamin B6 (Fig. S1). PL1 provided the means to label cells during active growth and metabolism, which was critical for the experiments to probe the 2AA damage. To make efficient use of the PL1 probe for *in vivo* labeling studies, the effect of a *ptsJ* mutation was assessed. PtsJ is a transcriptional repressor in *S. enterica,* and lesions in *ptsJ* result in an overexpression of *pdxK* (encoding PL/PN/PM kinase, EC 2.7.1.35) (26). Incorporation of the B6 vitamers (PL, PM, and PN) (Fig. 1B) and the PL probes (PL1 and PL2) requires phosphorylation by PdxK (25); thus, increased levels of PdxK potentially allow more efficient incorporation of PL compounds into cellular metabolism. However, deleting *ptsJ* had no effect on the minimal concentration of PL that was required to satisfy a *pdxJ* mutant (Fig. 2). Based on these and additional growth analyses, the *pdxJ* and *pdxJ ridA* mutant strains were used throughout the study without modification. Further, these studies established that 1 µM of PL or PL1 was sufficient to satisfy the growth requirement of *S. enterica*.

### *S. enterica* mutants differ in the ability to access PL1 as a source of B6

**Fig 3:**
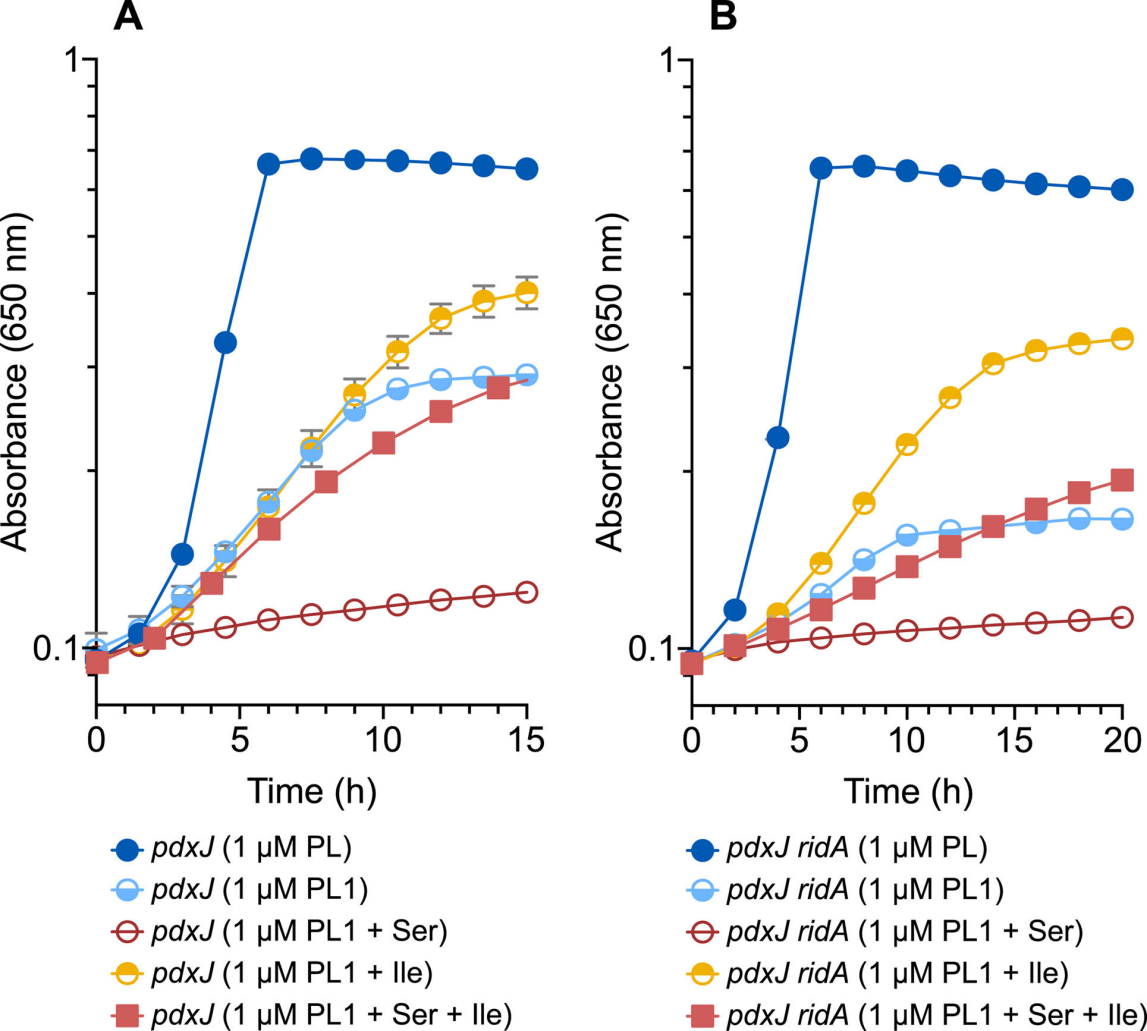
Mutants perturbed in PLP metabolism differentially salvage PL1. Two mutant strains that require exogenous B6 (*pdxJ* [**A**] and *pdxJ ridA* [**B**]) were grown in minimal glucose (11 mM) medium with the indicated additions. Growth was determined by monitoring the optical density at 650 nm of *n* = 3 biological replicates over time, with the mean plotted. Strains are designated by the symbols in the included legend. PL = pyridoxal, Ser = serine, and Ile = isoleucine.

The ability of *S. enterica* to use the PL1 probe as the sole source of vitamin B6 demonstrated that cells can transport, phosphorylate, and incorporate PL1(P) into proteins to generate active PLP-DEs. The data that were obtained during growth optimization indicated differences in the metabolism of PL1 compared to that of PL. Our interest in 2AA stress, particularly its relationship to PLP salvage and homeostasis, prompted us to analyze the growth in different nutrient conditions. Based on the growth analyses, a *pdxJ* mutant could salvage PL1 with different efficiency depending on the composition of the medium (Fig. 3A). Significantly, the growth with PL1 as the sole source of vitamin B6 was compromised by the presence of serine. This growth was completely restored by the addition of isoleucine. In contrast, growth in the presence of PL was not impacted (data not shown), confirming that the salvage of the native B6 vitamer was not affected by the presence of these amino acids. The growth effects that were caused by serine and isoleucine suggested a connection to 2AA stress.

A strain lacking RidA grew worse with PL1 as the sole source of vitamin B6 compared to the wild-type parental strain in minimal medium, while PL allowed full growth of both strains (Fig. 3B). Differences in the phenotype of the strains with and without RidA have been attributed to the accumulation of 2AA, which occurs in the latter (10, 13). The significant growth stimulation, which was afforded by providing isoleucine, supports the hypothesis that a component involved in PL1 salvage is negatively impacted by 2AA (Fig. 3B). Further, the addition of serine to the medium eliminated growth when salvage of PL1 was required, an effect that was also eliminated by the addition of isoleucine. As with the wild-type, the growth was not impacted by the addition of these amino acids to the medium when PL was the sole source of vitamin B6 (Fig. S5). These results further supported a connection to 2AA stress since serine increases the level of this stress in the cell, particularly in a *ridA* mutant, and isoleucine limits the amount of 2AA by allosterically inhibiting the primary 2AA generator, IlvA (Fig. 1) (6, 10). Together, these data suggest that the salvage and/or incorporation of PL1 is sensitive to the level of 2AA in the cell.

### PLP-DEs and PLP-associated proteins are enriched with PL probes in *Salmonella enterica*

**Fig 4:**
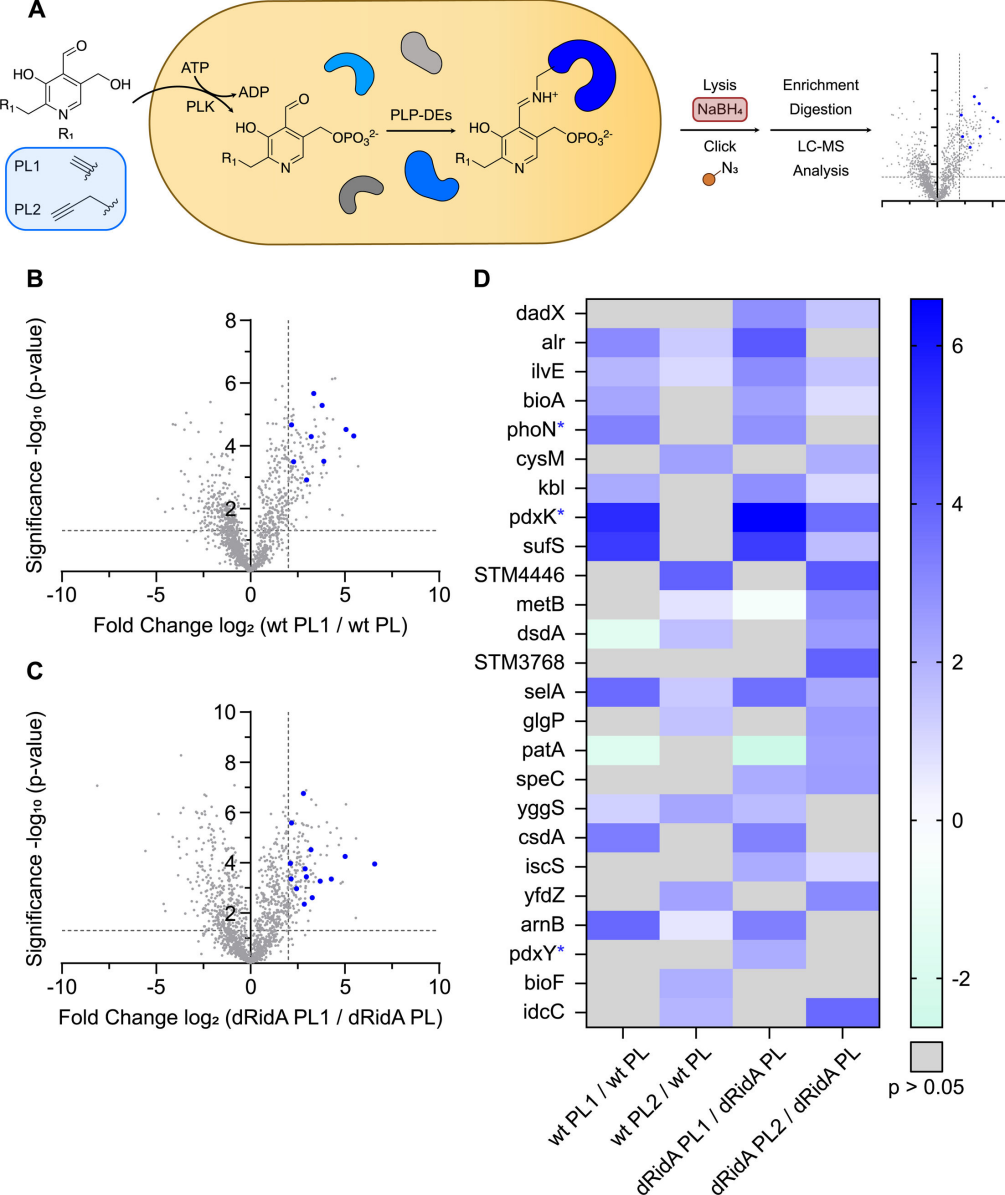
Profiling PLP-DEs in *S. enterica* with PL1 and PL2 probes after NaBH_4_ reduction (workflow #1). (A) Schematic representation of *in situ* PLPome mining with PL1 and PL2 probes in *S. enterica*. After cellular uptake, probes are phosphorylated and incorporated into PLP-DEs. Cells are lysed, and a stable Schiff base between the PL mimics and PLP-DEs is built upon reduction with NaHB_4_. PL mimics bearing an alkyne functionality are clicked to biotin azide for subsequent streptavidin enrichment, following tryptic digestion and LC-MS/MS analysis. (**B** and **C**), Volcano plots of *S. enterica* wild-type (wt) (**B**) or cells lacking RidA (dRidA) (**C**) grown with PL1 (1 µM) compared to those grown with PL (1 µM) as the sole source of B6 in minimal medium. A two-sample Student’s *t*-test was performed for all relevant comparisons to calculate the fold change values and statistical significance. The vertical and horizontal dashed lines represent a log_2_ fold enrichment ratio of 2 and a -log_10_ *P*-value of 1.3 (*p*-value of 0.05), respectively. Significantly enriched PLP-DEs are indicated as blue dots. (**D**) Heatmap showing log_2_ fold enrichment ratios of significantly enriched PLP-DEs with PL1 and PL2 probes in wild-type and dRidA cells. Gene names of proteins with a noncanonical association with PL(P) are labeled with a blue asterisk. The data represent *n* = 4 biologically independent replicates. PL = pyridoxal, PLK = pyridoxal kinase, PLP-DEs = pyridoxal phosphate-dependent enzymes, ATP = adenosintriphosphate, ADP = adenindiphosphate, and wt = wild-type.

For simplicity and to emphasize the relevant variables, the *pdxJ* and *pdxJ ridA* mutant strains are designated throughout as WT and dRidA, respectively. These two strains were labeled with each of the two PL probes to enrich PLP-DEs using a chemical proteomics workflow that included a reduction step with sodium borohydride (NaBH_4_) (workflow #1, Fig. 4A). In the case of PL1, the labeling workflow involved growing the cultures in minimal medium, with PL1 (1 µM) provided as the sole source of vitamin B6. After 17 h of growth, the cells were harvested, and the cell mass was used for analyses. Control samples were generated from cultures that were grown with PL (1 µM) provided as the sole source of vitamin B6. Significantly, with this workflow, both PL and the PL1 probe are taken up, phosphorylated, and incorporated into relevant enzymes by the salvage enzymes of *S. enterica*, as evidenced by the ability of PL1 to support growth (Fig. S1A) (25).

In contrast, a different approach was required with PL2 since this probe was unable to satisfy the vitamin B6 requirement for cellular growth (Fig. S1B) (25). To label cells with PL2, resting cells were incubated with the probe using a protocol previously used to efficiently enrich PLP-DEs in other bacteria (25). The ability to enrich PLP-DEs with a probe that is unable to support growth suggests PL2(P) populates the enzymes but does not support sufficient catalysis, by some or all the enzymes, to allow growth.

Volcano plots corresponding to the PL1 labeling data from both the WT and dRidA strains and a heatmap depicting the proteins that were enriched with either probes, when compared to the control, are shown in Fig. 4B to D, respectively. PLP-DEs were identified as a hit with a *P*-value of <0.05 and a log_2_ fold enrichment ratio of >2. Volcano plots displaying the PL2 labeling data from both WT and dRidA strains are shown in Fig. S2. In total, 25 proteins of interest were significantly enriched (Fig. 3D; Table S1), with a significant overlap between those labeled with the PL1 and PL2 probes. Twenty-two of the 25 enriched proteins are functionally assigned or predicted as PLP-DEs, and we considered that three proteins (PdxK, PdxY, and PhoN) are PLP-associated. In *S. enterica,* there are ~57 known or predicted PLP-DEs (27). With the use of two PL probes and two strains, 38% of the expected PLP-DEs were enriched with this workflow (workflow #1). These numbers represent a solid coverage for the use of both probes since each probe has a different labeling preference and thus expands the total number of enriched PLP-DEs (25). The subsequent analyses focused on the data obtained with the PL1-labeled cells as this allowed the salvage and incorporation of the probe to be mediated by growing cells, making the results physiologically relevant.

There was no clear difference in the enrichment pattern of PLP-DEs in the samples from the strains with or without the *ridA* mutation (Fig. 4D). This result was anticipated since the 2AA accumulation in a *ridA* mutant was not expected to impact the PLP occupancy of enzymes. In total, these data confirmed that PLP-DEs can be enriched in *S. enterica* with PL probes and showed that labeling with the PL1 probe in growing cells could be used to enrich for PLP-DEs.

In addition to the known or predicted PLP-DEs, a few proteins that are involved in PLP biosynthesis and/or salvage (PdxK and PhoN) were enriched with workflow #1 (Fig. 4A). PdxK is required to salvage unphosphorylated B6 vitamers (and PL probes) and is involved in the homeostasis of the vitamers (28–30). Both PL and PLP act as inhibitors of PdxK by forming a Schiff base with a lysyl residue near the active site (28, 29). This inhibition is proposed to play a physiological role in the bacterial cells by controlling the levels of free PLP, which can be toxic due to the reactive aldehyde (31, 32). The formation of a Schiff base has been demonstrated *in vitro* with purified PdxK (28, 29) and visualized in the crystal structure of PdxK from *S. enterica (*33). The results here support the association of PdxK with PLP, which was concluded based on the *in vitro* work. PhoN (nonspecific acid phosphatase, EC 3.1.3.2) was another protein with a noncanonical association with PLP, which was enriched with the PL1 probe (Fig. 4). PhoN is a periplasmic acid phosphatase present in *S. enterica*, but not in *E. coli*, and is necessary for *S. enterica* to utilize exogenous PLP as a sole source of vitamin B6 (34). When assessed *in vitro*, PhoN cleaves the phosphate from PLP to produce PL, which is presumed to pass through in the inner membrane and is phosphorylated by PdxK prior to being loaded into PLP-DEs (34). The ability of the PL mimics to enrich proteins that have an association with PL(P) metabolism but are not standard PLP-DEs suggests that the obtained data can be mined for insights on subtle aspects of the PLP metabolism.

**Fig 5:**
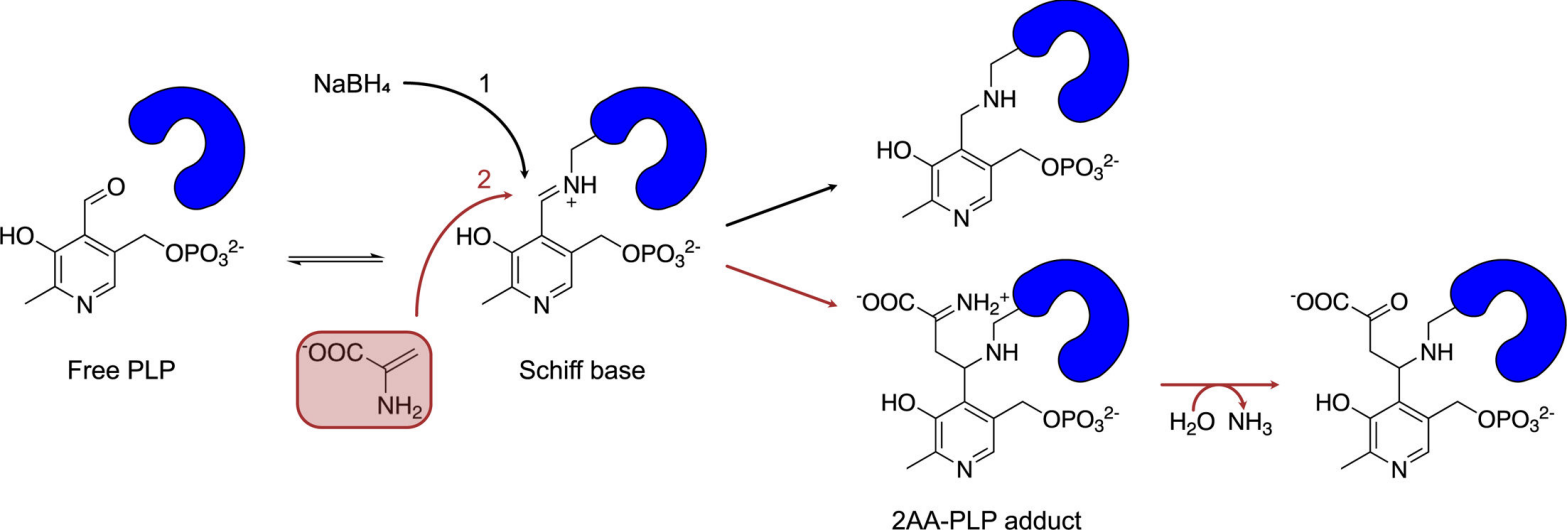
Associations of a PLP cofactor with PLP-DEs. Schematically shown are different associations that can exist between PLP and proteins. PLP binds to proteins via Schiff base (aldimine) formation. In the cell, this internal aldimine is in equilibrium with the free enzyme/free PLP. The equilibrium is dependent on the chemical environment of PLP and the stability it generates. The internal aldimine can be reduced by (1) sodium borohydride (NaBH_4_) to generate a stable covalent linkage between the lysyl residue of the protein and the cofactor (black arrows). Alternatively (2), 2-aminoacrylate (red) can attack the internal aldimine to form a stable covalent linkage of a 2AA-PLP adduct and the lysyl residue (red arrow). In the latter, the adduct can be deaminated by water to result in a protein-bound pyruvate-PLP adduct (red arrows). PLP = pyridoxal-phosphate, 2AA = 2 aminoacrylate, and PLP-DEs = pyridoxal phosphate-dependent enzymes.

Despite the diversity of reactions catalyzed by PLP-DEs, the catalytic mechanisms are unified by the involvement of an internal aldimine formed with a lysyl residue of the enzyme (Fig. 5). The resulting Schiff base linkage is a dynamic covalent bond that is reversible, with its rate determined by the chemical environment in which the cofactor resides. Thus, some enzymes are loosely associated with their PLP cofactor, and various treatments or manipulations can increase or decrease the loss of its cofactor. In workflow #1, the instability of a Schiff base is mitigated by reducing the internal aldimine with sodium borohydride to generate a covalent bond between PLP and its respective enzyme (Fig. 5). We assumed that in the absence of a reduction step with sodium borohydride, many, if not all, Schiff base linkages between PLP and the protein would be disrupted by the harsh urea treatment in the workflow.

### Many PLP-DEs and PLP-associated proteins are stably bound to PLP *in vivo*

**Fig 6:**
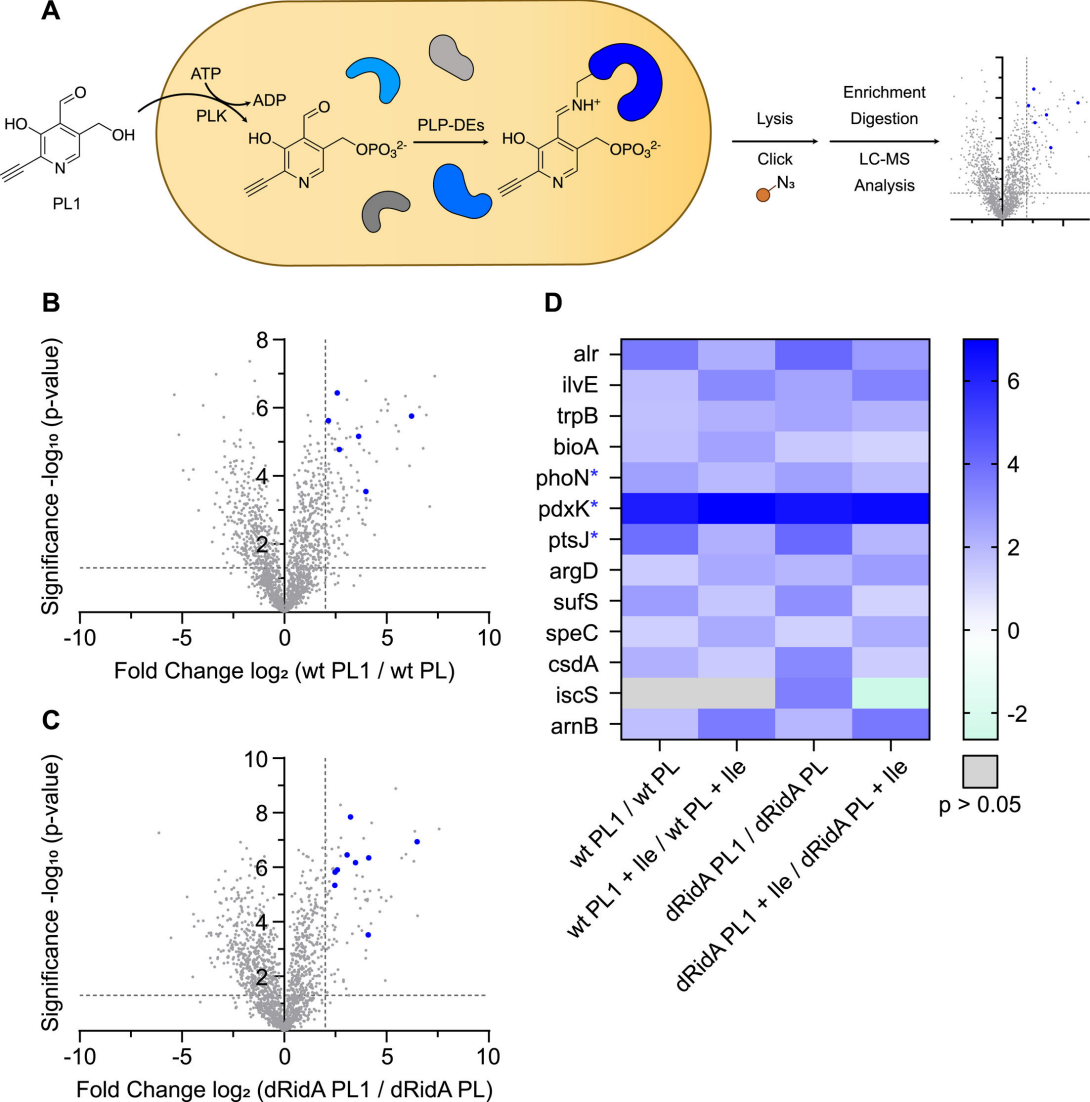
Profiling PLP-DEs in *S. enterica* with PL1 without NaBH_4_ reduction (workflow #2). (**A**) Schematic representation of *in situ* PLPome mining with the PL1 probe in *S. enterica*. After cellular uptake, the probe is phosphorylated and incorporated into PLP-DEs. Cells are lysed, and the PL1 mimic bearing an alkyne functionality is clicked to biotin azide for subsequent streptavidin enrichment, following tryptic digestion and LC-MS/MS analysis. (**B** and **C**) Volcano plots of *S. enterica* wild-type (wt) (**B**) or cells lacking RidA (dRidA) (**C**) grown with PL1 (1 µM) compared to those grown with PL (1 µM) as the sole source of B6 in minimal medium. A two-sample Student’s *t*-test was performed for all relevant comparisons to calculate the fold change values and statistical significance. The vertical and horizontal dashed lines represent a log_2_ fold enrichment ratio of 2 and a -log_10_ *P*-value of 1.3 (*P*-value of 0.05), respectively. Significantly enriched PLP-DEs are indicated as blue dots. (**D**) Heatmap showing log_2_ fold enrichment ratios of significantly enriched PLP-DEs with the PL1 probe from wild-type and dRidA cells that were grown with or without the addition of isoleucine. Gene names of proteins with a noncanonical association with PL(P) are labeled with a blue asterisk. The data represent *n* = 4 biologically independent replicates. PL = pyridoxal, PLK = pyridoxal kinase, PLP-DEs = pyridoxal phosphate-dependent enzymes, ATP = adenosine triphosphate, ADP = adenosine diphosphate, Ile = isoleucine, and wt = wild-type.

In a next step, workflow #1 was modified to specifically enrich proteins that have a stably bound PLP (or PL1P) *in vivo*. For this, the sodium borohydride treatment was eliminated to allow dynamic covalent Schiff base linkages to dissociate without interference (workflow #2). We presumed that PLP-DEs that were enriched with workflow #2 would include both those with a covalent bond between the protein and PLP and those with a stable binding pocket for PLP. In a simple scenario, the proteins that were enriched in workflow #2 would be a subset of those of workflow #1. WT and dRidA strains were grown in two defined media using PL1 as the sole source of vitamin B6 (Fig. 6A). Volcano plots for two of the four tested conditions (Fig. 6B and C; Fig. S3) and a heatmap describing the fold enrichment for 13 PLP-DEs and PLP-associated proteins are shown (Fig. 6D; Table S2). In total, five proteins that were enriched with workflow #1 were not enriched with workflow #2, suggesting that the sodium borohydride reduction step was required to stabilize the interaction of these proteins with PLP. Two PLP-DEs, TrpB (tryptophan synthase, EC 4.2.1.122) and ArgD (n-acetylornithine aminotransferase, EC 2.6.1.11), were enriched in workflow #2, but not in workflow #1.

The protein that was most highly enriched across the four tested conditions was PdxK, which is not a canonical PLP-DE. The enrichment of PdxK stressed the stability of its association with PLP and supported the role of vitamin B6 binding in regulating PdxK activity *in vivo (*31, 32). In addition, the transcriptional repressor of vitamin B6 salvage, PtsJ, was enriched with workflow #2. It is an unusual MocR-like regulator that is found in *S. enterica* and is a transcriptional repressor of *pdxK* (26). PLP is an effector of PtsJ, which binds in the C-terminal domain of the protein that is structurally homologous to the fold type I family of PLP-DEs (35). The data showed that the binding of PLP was tight enough to be retained through the sample preparation in workflow #2.

### 2AA stress impacts the enrichment of PLP-DEs and PLP-associated proteins with PL1

**Fig 7:**
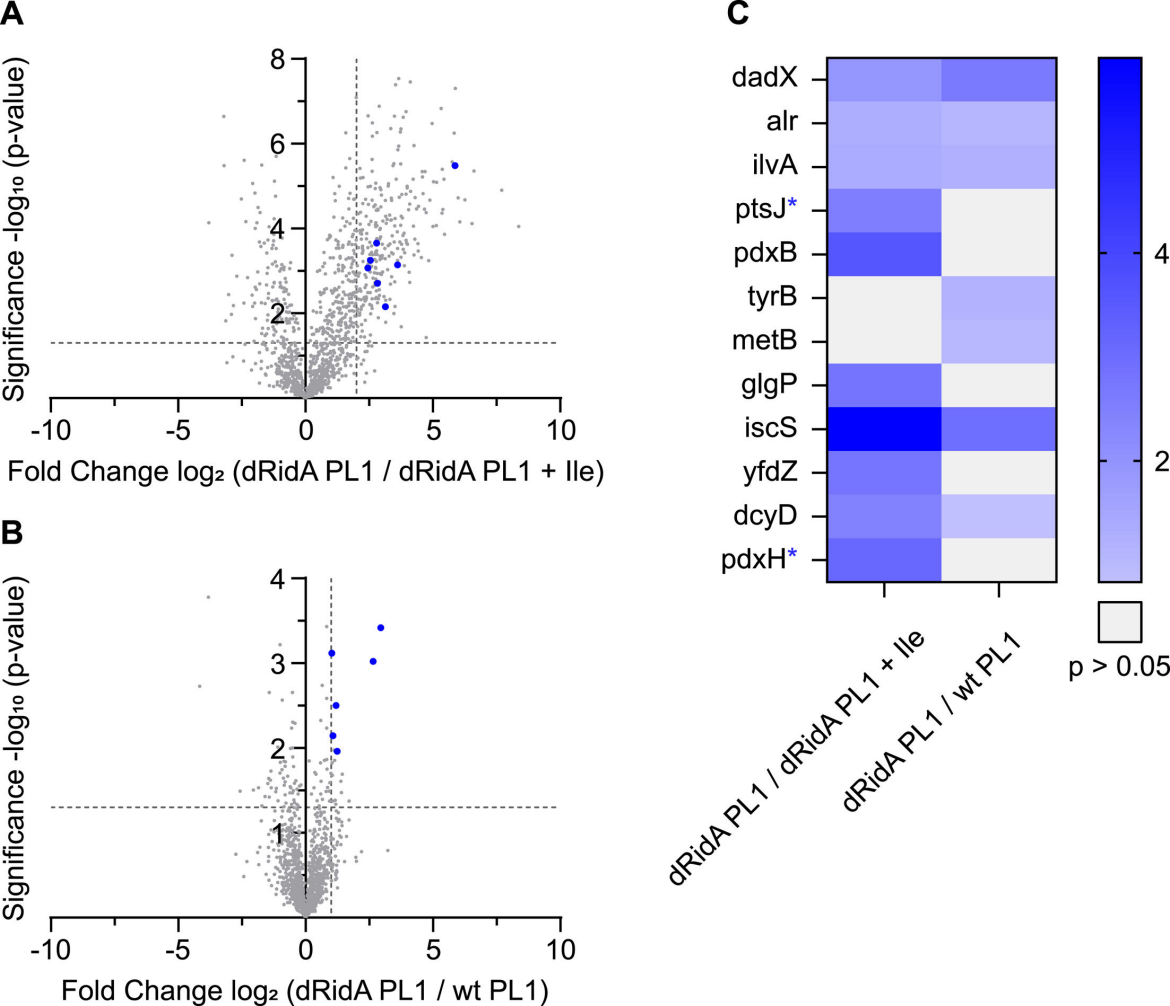
Profiling PLP-DEs in *S. enterica* with PL1 under 2AA stress. (**A**) Volcano plot of *S. enterica* cells lacking RidA grown with 1 µM PL1 as the sole source of B6 in minimal medium compared to the same condition supplemented with 0.3 mM isoleucine. (**B**) Volcano plot of *S. enterica* cells lacking RidA grown with PL1 (1 µM) as the sole source of B6 in minimal medium compared to wild-type grown in the same condition. A two-sample Student’s *t*-test was performed for all relevant comparisons to calculate the fold change values and statistical significance. The vertical and horizontal dashed lines represent a log_2_ fold enrichment ratio of 2 (**A**) or 1 (**B**) and a -log_10_ *P*-value of 1.3 (*P*-value of 0.05), respectively. Significantly enriched PLP-DEs are indicated as blue dots. (**C**) Heatmap showing log_2_ fold enrichment ratios of significantly enriched PLP-DEs with the PL1 probe. Gene names of proteins with a noncanonical association with PL(P) are labeled with a blue asterisk. The data represent *n* = 4 biologically independent replicates. PL = pyridoxal, Ile = isoleucine, and wt = wild-type.

The design of workflow #2 was motivated by a desire to understand how 2AA could impact the PLP-protein associations on a global scale. 2AA attacks the PLP cofactor in some PLP-DEs, resulting in a 2AA-PLP adduct that is irreversibly bound in the active site of the protein (Fig. 5) (18, 36). As such, 2AA stress compromises the activity of multiple enzymes and alters the metabolic network, often in subtle ways. Proteins that have been attacked by 2AA would be enriched in workflow #2 due to the irreversibly bound 2AA-PL1(P) adduct. However, there is no mechanism to separate the 2AA-damaged proteins from those that are enriched based on other features that stabilize the PLP-protein interaction. To focus specifically on the impact of 2AA, cells were exposed to different levels of 2AA stress and labeled with the PL1 probe. Specifically, dRidA cells grown in minimal medium with PL1 as the sole source of vitamin B6, a condition that mimics high 2AA stress, were compared to dRidA cells grown in minimal medium with PL1 as the sole source of vitamin B6 and isoleucine, which represents a condition of low 2AA stress. The addition of isoleucine to the medium inhibited the serine/threonine dehydratase IlvA, the major source of 2AA in the cell (1, 10) (Fig. 1). A second comparison between PL1-labeled samples from high and low 2AA conditions was obtained from WT and dRidA strains, when both were grown in minimal medium. In this medium, the dRidA strain accumulates more 2AA stress than the WT. In each case, the labeled samples were processed using workflow #2, and the log_2_ fold enrichment ratios of the identified PLP-DEs were determined. For the latter experiment, the cutoff of the log_2_ fold enrichment ratio was set to >1. The data are represented in volcano plots (Fig. 7A and B), and a heatmap that shows the PLP-DEs and PLP-associated proteins that are enriched in the presence of a higher 2AA stress (Fig. 7C, and Table S3). Importantly, these analyses require that the strains are grown with the PL1 probe as the sole source of PL since the 2AA stress is generated during cell growth. Labeling protocols that depend on PLP exchange would not label proteins containing the 2AA-PL1(P) adduct and would thus not enrich the respective PLP-DEs since an exchange is not possible once the adduct is irreversibly bound to the protein.

With the analyses described above, twelve PLP-DEs or PLP-associated proteins could be significantly enriched in the presence of high 2AA stress and thus predict potential targets of 2AA (Fig. 7). Interestingly, only three of the proteins (Alr, PtsJ, and IscS) were enriched in the preceding experiment with workflow #2 when enrichment was defined by a ratio of PL1/PL labeling (Fig. 6; Fig. S4; Table S2). Conversely, the remaining nine proteins were only differentially enriched with the PL1 probe in response to high 2AA stress. Three of the proteins (Alr, IscS, and DadX) were also enriched in the applied conditions with a reduction step (workflow #1) (Fig. 4; Fig. S4; Table S1). The data depicted in Fig. 4 and 6 show PLP-DEs that were labeled with PL1 and enriched by comparing to PL-labeled samples as a control. In contrast, the data in Fig. 7 show the enriched PLP-DEs under high 2AA stress.

Not all characterized 2AA target proteins were detected as such in this global analysis. For instance, IlvE (branched-chain amino acid aminotransferase, EC 2.6.1.27), AspC (aspartate aminotransferase, EC 2.6.1.1), and GlyA (serine hydroxymethyltransferase, EC 4.3.1.18) are targeted by 2AA when their activity is measured (6, 16, 19) or the protein is individually analyzed (18). This result emphasizes that a global proteome analysis with a single PL probe results in an underestimate of the 2AA-targeted proteins in *S. enterica*. Satisfyingly, several PLP-DEs targeted by 2AA *in vitro* were among the proteins enriched in response to high 2AA stress: DadX (alanine racemase, catabolic, EC 5.1.1.10), Alr (alanine racemase biosynthetic, EC 5.1.1.10), IscS (cysteine desulfurase, EC 2.8.1.7), and IlvA (threonine deaminase, EC 4.3.1.19) (17, 19, 25). Enrichment of IlvA was noteworthy since this enzyme is the major source of 2AA in multiple organisms (13). This protein was assumed to be resistant to 2AA damage, an assumption that was initially supported by the finding that the IlvA activity is not decreased in a *ridA* mutant (6). However, a study that investigated the modification of specific overexpressed and purified proteins confirmed that a 2AA-PLP adduct was associated with IlvA, which was purified from cells undergoing high 2AA stress (18). Importantly, the study concluded that the adduct was not formed from the 2AA generated in the IlvA active site, but rather from the free pool of 2AA that accumulated in a *ridA* mutant background (18). IscS (cysteine desulfurase, EC:2.8.1.7) had the highest log_2_ fold enrichment under high 2AA stress. 2AA-dependent damage to IscS has clear metabolic implications in the organisms queried thus far, and it is considered the primary target of 2AA (24). IscS is a critical component of Fe-S cluster biogenesis in numerous organisms and the only enzyme to date for which 2AA-resistant variants exist (24). Two additional enzymes, TyrB (aromatic amino acid aminotransferase, EC 2.6.2.67) and YfdZ (alanine transaminase, EC 2.6.1.2), that catalyze transaminase reactions and are expected to be damaged by 2AA were enriched in response to high 2AA stress. Three additional 2AA-dependent proteins were significantly enriched under high 2AA stress, although they do not have a known association with PLP metabolism: DcyD (D-cysteine desulfhydrase, EC 4.4.1.15), MetB (*O*-succinylhomoserine(thiol)-lyase, EC 2.5.1.48), and PdxB (4-phosphoerythronate dehydrogenase, EC 1.1.1.29). The remaining proteins that were enriched in the presence of 2AA stress are not canonical PLP-DEs but have a described association with PLP (GlgP, PdxH, and PtsJ). GlgP (glycogen phosphorylase, EC 2.4.1.1) possesses a PLP molecule in its active site (37) and uses the phosphate group of PLP, rather than the standard internal aldimine in the catalytic reaction. The enhanced enrichment of this protein in the presence of high 2AA levels suggests that an attack by 2AA would increase the pool of this enzyme with a covalent association with PLP. The 2AA-dependent enrichment of PdxH (PN/PL oxidase, EC 1.4.3.5) and PtsJ was not anticipated since neither of these proteins catalyzes a PLP-dependent reaction, but both bind and are regulated by PLP. Interestingly, PLP is the product of PdxH catalysis and has been shown to associate with and inhibit the enzymatic activity (38). The 2AA-dependent enrichment of PLP-DEs and PLP-associated proteins here may suggest new physiological features of these enzymes.

### Conclusions

A chemical proteomics workflow to enrich PLP-DEs using a click chemistry-based protocol (25), designated as workflow #1 herein, was combined with a modified workflow #2 to investigate the questions of PLP metabolism in *S. enterica*. In workflow #1, strains of *S. enterica* unable to synthesize PLP were labeled with each of two pyridoxal probes PL1 and PL2 following a reduction step with sodium borohydride (25). When provided as the sole source of PLP, PL1 but not PL2, allows growth of *S. enterica,* as it does for *E. coli* (25). For these experiments, cells were grown with PL1 as the sole source of PL, ensuring that this probe was salvaged, phosphorylated, and loaded into PLP-DEs by the cellular machinery that salvages PL. In contrast, nongrowing cells were labeled with PL2. The presumption is that PL2(P) enters relevant enzymes by PLP exchange. After labeling the two strains with PL1 and PL2, 25 of the 57 presumed PLP-DEs in *S. enterica* could be significantly enriched. The distribution and overlap of PLP-DEs that were enriched by the two probes justify the use of cofactor exchange to capture PLP-DEs. However, more extensive studies are needed to determine whether cofactor exchange can detect all PLP-DEs. It is possible that there are enzymes, perhaps those with a strong affinity for PLP, that are not labeled in the absence of protein synthesis and turnover and thereby underrepresented by this method. Further, the comparison herein was made in cells with wild-type, functioning salvage enzymes, and it remains to be seen if the labeling is affected when the PLP metabolism is altered.

In workflow #2, the reduction step with sodium borohydride was eliminated to better understand the state of PLP-protein interactions that exist in the cell. We assumed that workflow #2 would enrich PLP-DEs with a stabilizing environment for the Schiff base linkage or an irreversible covalent linkage between PLP and the protein. The obtained data were consistent with this assumption, and 13 proteins that were PLP-dependent or -associated were significantly enriched. The most highly enriched protein with workflow #2 was PdxK, a protein with a noncanonical association with PLP. PdxK phosphorylates PL (and PL1) to generate PL(1)P**,** which forms a Schiff base with the enzyme *in vitro*. The results here support the conclusion that a stable association of PdxK with PLP also exists *in vivo*.

A primary goal of this study was to expand our understanding of 2AA stress by identifying the breadth of proteins with a covalently bound 2AA-PLP adduct under 2AA stress. We anticipated such proteins would (i) be enriched in the absence of a reduction step and (ii) be significantly enriched under high 2AA stress. In this analysis, the relevant comparison was made between samples that were labeled with PL1 in two different conditions (high and low 2AA stress), rather than involving a PL control. In this way, we specifically addressed the level of 2AA as the relevant variable. Twelve proteins were significantly enriched and met these criteria and were presumed to be targets of 2AA. Several of these proteins were targeted by 2AA in previous studies. This result increased confidence that, with refinement, the proteomic approach implemented here has the potential to detect a global signature of 2AA stress in *S. enterica* and other organisms. In addition to PLP-DEs, two proteins with noncanonical associations with PLP were enriched in the presence of high 2AA stress. Of particular interest were PdxK and PdxH, both of which have roles in salvage and homeostasis of the B6 vitamer pool, and whose direct association with PLP has been postulated but not characterized *in vivo*. The results here warrant further studies to better define the association of PdxK and PdxH with PLP and the possibility that 2AA stress impacts that association.

The ability of *S. enterica* to efficiently salvage PL1 as the sole source of vitamin B6 was impacted by the genetic background and growth medium. Salvage of natural PL was not affected by these parameters, indicating that the PL1 probe was sufficiently different compared to PL to perturb the process(es) involved in PL salvage from the environment. These observations provide an exciting new avenue to probe 2AA stress and the mechanism(s) used by *S. enterica* to salvage and incorporate vitamin B6 from the environment.

In summary, the work herein generated insights into 2AA stress on a global scale. This work emphasizes that combining global approaches with physiological insights from genetic and mechanistic studies enhances our understanding of the complex integration of metabolic networks in a living organism.

## MATERIALS AND METHODS

### Media and quantification of bacterial growth

All strains used in this study are derivatives of *Salmonella enterica* serovar Typhimurium strain LT2 (*S. enterica*) and are part of the Downs laboratory collection. In all cases, mutant gene alleles indicated (i.e., *ridA*) are loss-of-function mutations. Rich medium used was Difco Nutrient Broth (NB; 8 g/L) with NaCl (5 g/L). Minimal medium was no-carbon E salts (NCE) (39), supplemented with trace elements (40), MgSO_4_ (100 µM), and glucose (11 mM) as the sole carbon source. Nutritional supplements (i.e., B6 vitamers) and their concentrations are noted in the text. Both pyridoxal probes PL1 and PL2 were synthesized as previously reported (25) and were available in the Sieber laboratory. Other chemicals were purchased from Millipore-Sigma.

Growth of *S. enterica* strains was determined quantitatively in liquid medium. Strains were grown overnight in 2 mL NB. Cells from overnight cultures were pelleted at 3,200 × *g* for 15 minutes and resuspended in an equal volume of 0.85% saline prior to subculturing (2.5% inoculum) into the indicated medium (200 µL total volume). Growth was measured via optical density at 650 nm (OD_650_) in a 96-well plate using a BioTek ELx808 plate reader (BioTek Instruments, Winooski, VT). All graphs were created using Prism 9.5.1 (GraphPad Software, La Jolla, CA). Typically, there were three biological replicates, and growth was plotted with error bars.

### Sample preparation

#### Labeling with PL2

Four independent cultures of the strain DM15906 (*pdxJ662::Km*) and DM17667 [*pdxJ662::Km ridA1::Tn10d*(*Tc*)] were grown in NB medium overnight at 37°C with shaking. Fully grown cultures were centrifuged, and each pellet was resuspended in 0.5 mL saline. An aliquot of each cell resuspension (0.2 mL) was used to inoculate 10 mL minimal medium containing 1 µM pyridoxal to ensure PLP generation, 5 mM L-serine to induce 2AA production, and 1 mM glycine and 0.1 mM thiamine to enhance growth. Cultures were grown with shaking at 37°C for 7–8 h to reach full density (OD_600_ = 1.44–1.50). From each culture, two 4 mL aliquots were centrifuged (6,000 × *g*, 4°C, 10 min). Each cell pellet was resuspended in 0.5 mL minimal medium and transferred into 1.5 mL epi tubes and pelleted. The final cell pellets were resuspended in 0.2 mL minimal medium with or without 100 µM PL2 and incubated for 2 h at 37°C with shaking. Each 200 µL sample was centrifuged, the supernatant decanted, and the pellets stored at −80°C prior to use.

#### Labeling with PL1

Four independent cultures of the strain DM15906 (*pdxJ662::Km*) and DM17667 [*pdxJ662::Km ridA1::Tn10d*(*Tc*)] were grown overnight in NB medium overnight at 37°C with shaking. An aliquot of each culture was used to inoculate (1:20) nutrient medium and grown for 4–5 h. The cells in each culture were pelleted and resuspended in saline. For each replicate, 5 mL of the cell suspension was inoculated into each of two flasks with 195 mL (2.5% [vol/vol]) minimal NCE medium supplemented with 1 mM MgSO_4_, trace metals, 11 mM glucose as the sole carbon source, and 1 µM PL1. One flask from each replicate was supplemented with 0.3 mM isoleucine, resulting in 16 samples. The 200 mL cultures were grown shaking in an Innova shaker overnight (16–20 h) at 37°C, 180 rpm.

Control samples were generated by using the above saline suspensions to inoculate each of two tubes with 20 mL (1% [vol/vol]) minimal NCE medium supplemented with 1 mM MgSO_4_, trace metals, 11 mM glucose as the sole carbon source, and 1 µM PL. The OD_650_ of PL samples and PL1 was measured. PL-supplemented cultures all grew to full density (OD_650_ ~ 1.3) in 16 h; with PL1-supplemented cultures, those with isoleucine grew to OD_650_ 0.5–0.6 in ~18 h, and those without Ile grew to OD_650_ ~ 0.2 in 20–22 h. Cells from all cultures were pelleted, and the bacterial cell pellets were stored at −80°C until use.

In total, two strains were grown with either PL or PL1 as the sole source of vitamin B6 under two conditions, minimal medium with and without supplementation of isoleucine (0.3 mM).

### Lysis and click chemistry treatment of labeled cells

Cell pellets were lyzed in 200 µL 0.5% SDS and 1% Triton-X 100 in PBS by sonication for three times for 15 s at 80% intensity (Sonopuls HD 2070 ultrasonic rod, Bandelin electronic GmbH). Cell debris was removed by centrifugation (at least 30 min at 21,000 × *g*, room temperature [RT]), and the clear lysate was transferred into new LoBind tubes. For the reduction step, 100 µL of each sample was treated with 10 mM NaBH_4_ in 0.1 M NaOH for 30 min at RT, and proteins were precipitated with ice-cold acetone for at least 4 h. Afterward, the proteins were pelleted by centrifugation (10 min at 21,000 × *g* and 4°C) to remove all soluble chemicals. The pellets were washed twice with 500 µL ice-cold MeOH by mild sonication for 10 s at 10% intensity and dissolved in 100 µL PBS. The protein concentration of all samples (nonreduced and reduced) was calculated by BCA assay (Roti Quant, Roth) and adjusted to 45 µL 2.23 mg/mL in a 96-well plate (polypropylene, V-bottom, Greiner cat. 651201). Each sample was treated with a click mix (0.6 µL 20 mM biotin-azide in DMSO, 2.5 µL 1.67 mM tris(benzyltriazoylmethyl)amine (TBTA) in 80% *t*BuOH and 20% DMSO, 1.2 µL 50 mM CuSO_4_ in H_2_O, and 0.6 µL 100 mM tris(2-carboxyethyl)phosphine (TCEP) in H_2_O) and incubated for 90 min at 950 rpm and RT. Then, 65 µL 8 M urea with 10 mM TCEP and 20 mM iodoacetamide (IAA) in H_2_O was added and incubated for 15 min at 950 rpm and RT to quench the reaction. Excess IAA was quenched by adding 2 µL 500 mM DTT.

### Enrichment and digestion

The following steps were performed with MS-grade reagents and a magnetic bead enrichment protocol as described previously (41, 42). In short, 10 µL of a double-concentrated 1:1 mixture of hydrophobic and hydrophilic carboxylate-coated magnetic beads (Cytiva, cat# 65152105050250 and 45152105050250), previously washed with H_2_O, was added to each sample. Next, proteins were precipitated onto the beads with 175 µL EtOH. All following steps were done with an automated liquid handling system (Hamilton Microlab Prep). Each washing step was performed by adding the respective washing solution to the beads, following shaking for 1 min at 800 rpm and RT, and incubation onto a 96-well ring magnet (Alpaqua, Magnum FLX) for 90 s before the supernatant could be removed (20 µL/s) again. Each sample was washed three times with 180 µL 80% EtOH and one time with 180 µL acetonitrile. The proteins were then eluted from the beads with 75 µL 0.2% SDS in PBS for 5 min at 800 rpm and 40°C. This step was performed twice to yield a total volume of 150 µL of the eluted proteins. Enrichment was accomplished by adding 50 µL streptavidin magnet beads (New England Biolabs, cat# S1420S), previously washed with 0.2% SDS in PBS, following incubation for 1 h at 800 rpm and RT in a plate shaker with a heated lid (ThermoMixer C, Eppendorf). For this, the plate was tightly sealed. Afterward, the beads were washed three times with 180 µL 0.1% NP-40 in PBS, twice with 180 µL 6 M urea, and three times with 200 µL H_2_O. Afterward, 100 µL 50 mM TEAB supplemented with 1 µL trypsin (ratio trypsin/protein 1:100, 0.5 µg/µL, sequencing grade, Promega) was added to each sample, the plate sealed again, and incubated overnight at 800 rpm and 37°C in a plate shaker with a heated lid. Peptides were eluted from the beads with 50 µL 3% FA for subsequent desalting with pre-equilibrated stage tips (two layers of styrenedivinylbenzene-reverse-phase sulfonate SDB-RPS (Empore, 3M) disks), as previously described (Coscia, 2020). Equilibration was done with 150 µL wash buffer 1 (1% [vol/vol] TFA in isopropanol) by centrifugation (20 min at 800 rpm and RT). Each sample was loaded (20 min at 500 × *g* and RT), followed by washing with 150 µL wash buffer 1 (20 min at 800 rpm and RT) and 150 µL wash buffer 2 (0.2% [vol/vol] TFA in H_2_O) (20 min at 800 rpm and RT). Elution was done with 50 µL elution buffer (1% ammonia; 80% acetonitrile) (15 min at 300 × *g* and RT). Peptides were dried in a centrifugal evaporator (Concentrator Plus, Eppendorf) and dissolved in 35 µL 1% FA. Each sample was subjected to LC-MS/MS measurement on Orbitrap Fusion or Orbitrap Eclipse Tribrid mass spectrometer (Thermo Fisher Scientific) and analyzed in the data-dependent acquisition (DDA; Fusion) or data-independent acquisition (DIA; Eclipse) mode.

### LC-MS/MS measurements on Orbitrap Fusion and Eclipse Tribrid instruments

#### Orbitrap Fusion

MS analysis was performed on an Orbitrap Fusion mass spectrometer coupled to an Ultimate3000 nano-HPLC via a Nanospray Flex Ion Source (ThermoFisher Scientific). Samples were loaded on the trap column (AcclaimPepMap 100 C18 (75 µm × 2 cm) trap/ flow rate: 5 µL/min, 0.1% TFA) before being separated on the Aurora Ultimate columns (2nd generation, 75 µm × 25 cm, ionopticks). Both columns were constantly heated to 40°C. For separation, a buffer B gradient (0.1% formic acid in acetonitrile) with a flow rate of 400 nL/min was applied (5–22% buffer B for 112 min, to 32% buffer B in 10 min, to 90% buffer B in 10 min, hold for 10 min, to 5% buffer B in 0.1 min, and hold 5% buffer B for 9.9 min). The Orbitrap was operated in a cycle time (3 s) data-dependent mode. An AGC target of 2e5, a maximum injection time of 50 ms, 60% RF lens, and a resolution of 120,000 in a scan range of 300–1,500 *m/z* in the profile mode was used. Monoisotopic precursor selection and dynamic exclusion (60 s) were turned on. For fragmentation, the most intense precursors with charges of 2–7 and intensities greater than 5e3 were chosen. Quadrupole isolation was performed using a range of 1.6 *m/z*. Precursor ions were separated using an AGC target of 1e4 and a maximum injection time of 100 ms. Fragmentation was performed using higher-energy collisional dissociation with a collision energy of 30%. Fragments were detected in the ion trap operating at a rapid scan rate. Data acquisition was done using Xcalibur software.

#### Orbitrap Eclipse Tribrid

The samples were analyzed via HPLC-MS/MS using a Vanquish Neo UHPLC (Thermo Fisher) equipped with a PepMap Neo 5 µm C18 300 µm × 5 mm Trap Cartridge (Thermo Fisher Scientific) and Aurora Ultimate separation columns (3rd generation, 20 cm nanoflow UHPLC compatible, ionopticks) with a Nanospray Flex Ion Source (ThermoFisher) coupled to an Orbitrap Eclipse Tribrid instrument (Thermo Fisher). Vanquish Neo UHPLC was operated in the Trap-and-Elute-Injection mode. Samples were loaded onto the trap column, and the subsequent separation was carried out with a flow rate of 400 nL/min using buffer A (0.1% FA in water) and buffer B (0.1% FA in MeCN). The separation column was heated to 40°C. Analysis started with a gradient from 5% to 22% buffer B for 30 min, a second gradient from 22% B to 32% B within 5 min, and a final increase to 90% B in 0.1 min. Isocratic washing with 90% B was performed for 9.9 min. For wash and equilibration, the following settings at the Vanquish Neo system were applied: 5% B, for separation column: fast equilibration was enabled, equilibration factor was set to 3; for Trap column: Fast Wash and Equilibration and Zebra Wash was enabled (Zebra Wash Cycles 2, equilibration factor set to automatic). The Orbitrab Eclipse mass spectrometer was run in a data-independent mode with a gradient time of 45 min and default charge state set to 2. Internal real-time mass calibration was performed using user-defined lock mass (*m*/*z* = 445.12003, positive). Master MS^1^-Scans were collected in the orbitrap in a scan range of 400–1,000 *m/z* at a resolution of 60,000. The RF-lens was set to 30% and with an AGC target set to standard with 100 ms maximum injection time. For DIA, ions in different *m/z* ranges (windows overlapping by 1 *m/z* from 399.5 to 1,000.5 *m/z*, see Table S4) were selected for MS^2^ scan, with isotope and dynamic exclusion (exclusion duration: 30 s) enabled. DIA MS^2^ spectra were collected with a scan range of 145–1450 *m/z* at a resolution of 15,000 with a normalized AGC target of 2,000% and a maximum injection time of 40 ms. Isolation was conducted in the quadrupole mode using a window of 1.6 *m/z*. Fragments were generated using higher-energy collision-induced dissociation (HCD, normalized collision energy: 30%) and finally detected in the orbitrap. Data were acquired using Thermo Scientific Foundation software version 3.1sp9 and Xcalibur version 4.6. The experiments were performed in four independent replicates.

### Data analysis of LC/MS-MS measurements

#### Orbitrap Fusion samples

Raw files acquired in the DDA mode were analyzed with MaxQuant (43) (version 2.1.0.0) with Andromeda search engine. The parameters can be found attached in the supplemental material (see Table S5). In brief, cysteine carbamidomethylation was set as a fixed modification and methionine oxidation and *N*-terminal acetylation as variable modifications. Trypsin (without *N*-terminal cleavage to proline) was set as the proteolytic enzyme with a maximum of two allowed missed cleavages. The label-free quantification (LFQ) mode (6) was performed with a minimum ratio count of 2. The “match between runs” (0.7 min match and 20 min alignment time window), second peptide identification, and iBAQ options were activated. Peptides were searched against the UniProt database for *S. typhimurium LT2* (taxon identifier: 99287 (UP000001014), downloaded 27 February 2023) (44). All other parameters were used as pre-set in the software. LFQ intensities were further processed with Perseus version 2.0.10.0 (45). Putative contaminants, reverse hits, and proteins identified by site only were excluded. LFQ intensities were log_2_-transformed and filtered to have at least three valid values in at least one group. Missing values were imputed based on a normal distribution (width = 0.3, down-shift = 1.8). A two-sample Student’s *t*-test was performed for all relevant comparisons. Hits with a *P*-value < 0.05 and a Student’s *t*-test difference of at least 1 were considered significant. For data visualization, a scatterplot [*x*-axis: Student’s *t*-test difference (probe/control); *y*-axis: -log Student’s *t*-test *P*-value (probe/control)] was created based on two-tailed Student’s *t*-test (FDR = 0.05). Graphs were plotted using GraphPad Prism 10.01.

#### Orbitrap Eclipse Tribrid samples

Raw files acquired in the DIA mode were analyzed with DIA-NN (version 1.8.1) using the library-free mode (46). The UniProt reference proteome of *S. typhimurium* LT2 (taxon identifier: 99287 (UP000001014), downloaded 27 February 2023) was used for library generation (44). The settings for precursor ion generation were the following. Library generation and deep-learning-based prediction of spectra, retention times, and ion mobilities were performed; trypsin/P was used as the protease with a maximum of two missed cleavages; protein N-terminal methionine excision was enabled; carbamidomethylation of cysteine was set as a fixed modification with no variable modifications; peptide lengths ranged from 7 to 30 amino acids; precursor charges ranged from 2 to 4; precursor *m/z* ranged from 300 to 1,800, and fragment *m/z* ranged from 200 to 1,800 for TIMS data. The precursor false discovery rate (FDR) was set at 0.01. Mass accuracy, MS1 accuracy, and scan window were all set to 0. Isotopologues, match between runs (MBR), and removal of likely interferences were enabled. A neural network classifier operated in the single-pass mode, and protein inference was performed at the gene level with heuristic protein inference enabled (--relaxed-prot-inf). The quantification strategy was set to robust LC (high precision), with cross-run normalization being dependent on retention times. Library generation employed smart profiling, with settings optimized for speed and RAM usage. LFQ quantities were extracted from the protein groups (pg) results file and were further analyzed with Perseus software (version 2.0.10.0) (45), as described above. LFQ intensities were log_2_ transformed. Subsequently, rows were annotated in respective groups and filtered based on three valid values in at least one group. A two-sample Student’s *t*-test was performed for all relevant comparisons. For data visualization, a scatterplot (*x*-axis: Student’s *t*-test difference [probe/control]; *y*-axis: -log Student’s *t*-test *P*-value [probe/control]) was created based on two-tailed Student’s *t*-test (FDR = 0.05). Graphs were plotted using GraphPad Prism 10.01.

There was no significant difference observed between DDA and DIA approaches as the target proteins are enriched with both acquisition modes. This highlights the strength and robustness of our approach.

## Data Availability

All data are available at request from the authors. The mass spectrometry proteomics data have been submitted to the ProteomeXchange Consortium through the PRIDE partner repository (47) (https://www.ebi.ac.uk/pride/) and are accessible using the data set identifier PXD060390.

## SUPPLEMENTAL MATERIAL

Supplemental Material msystems.00540-25-s0001.pdf Figures S1-S5 and Tables S1-S5.

ASM does not own the copyrights to Supplemental Material that may be linked to, or accessed through, an article. The authors have granted ASM a non-exclusive, world-wide license to publish the Supplemental Material files. Please contact the corresponding author directly for reuse.
