# Supplementary material for: Chemical proteomics enhances the understanding of 2AA stress in Salmonella enterica
Source: mSystems. 2025 May 29;10(6):e00540-25. doi: 10.1128/msystems.00540-25 (PMC12172467; doi:10.1128/msystems.00540-25)
Supplement: Supplemental Material — Figures S1-S5 and Tables S1-S5. [file msystems.00540-25-s0001.pdf]

SUPPLEMENTAL MATERIAL

Chemical Proteomics Enhances the Understanding of 2AA Stress in *Salmonella enterica*

Dominik Schum[#1], Michaela K. Fiedler[#1], Wangchen Shen[2,3], Stephan A. Sieber[1] and Diana M. Downs[2]*

[#] contributed equally

[1] School of Natural Sciences, Department of Bioscience, Chair of Organic Chemistry II, Center for Functional Protein Assemblies (CPA), Technical University of Munich (TUM), Ernst-Otto-Fischer Str. 8, Garching, 85748, Germany

[2] Department of Microbiology, University of Georgia, Athens, GA 30602 USA

[3] Current address: National Cancer Institute/NIH, Rockville MD 20850 USA

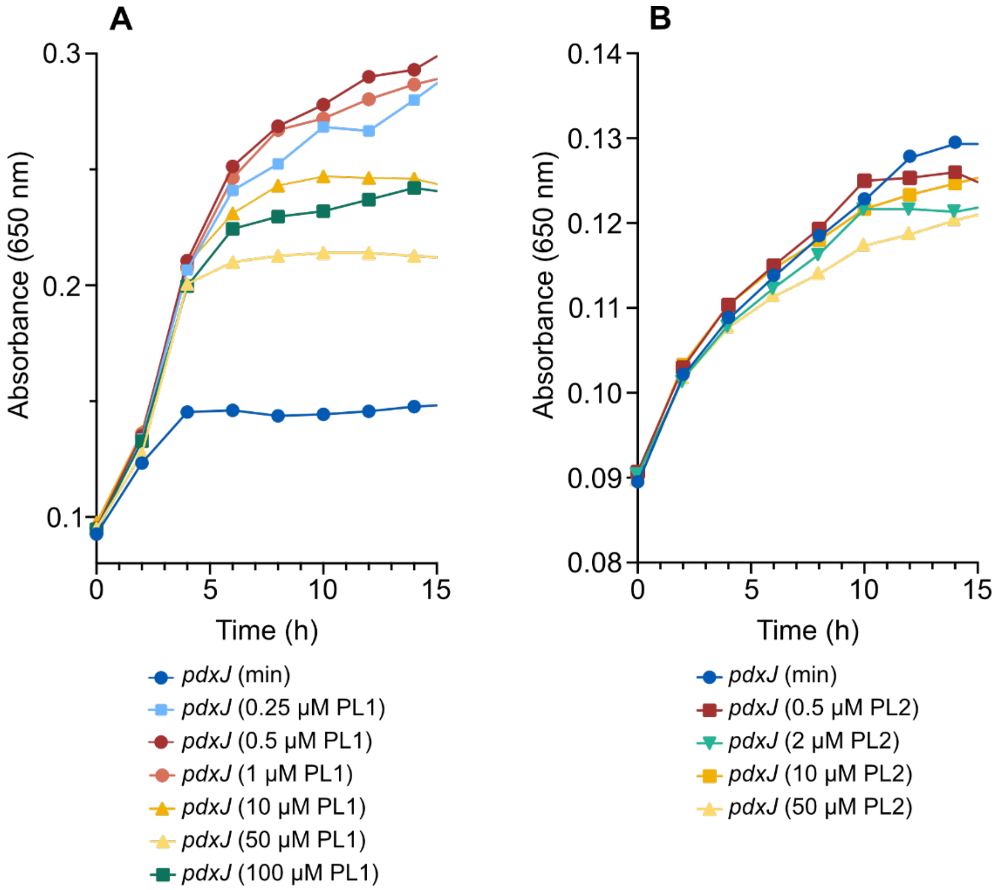

Figure S1. PL1, but not PL2 is used as sole source of B6 by *S. enterica*. A mutant strain that requires exogenous B6, *pdxJ*, was grown in minimal glucose (11 mM) medium with no addition (min) or with the indicated concentration of **PL1** (**A**) or **PL2** (**B**) in the medium. Growth was determined by monitoring optical density at 650 nm of n = 3 biological replicates over time, with the mean plotted. Strains are designated by the symbols in the included legend.

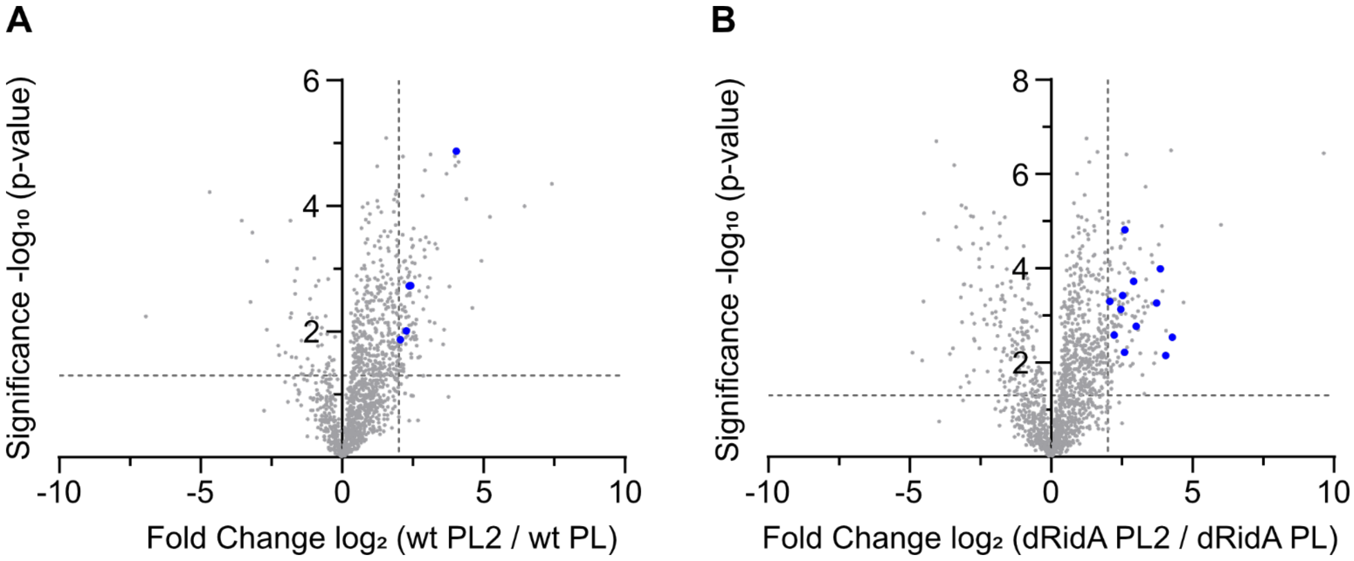

Figure S2. Profiling PLP-DEs in *S. enterica* with PL2 probe after $NaBH_4$ reduction (workflow #1). A and **B**, Volcano plots of *S. enterica* wildtype (A) or cells lacking RidA (dRidA) (B) incubated with **PL2** (100 µM) compared to those incubated with PL (1 µM) as the sole source of B6 in minimal medium. A two-sample Student's *t*-test was performed for all relevant comparisons to calculate the fold change values and statistical significance. The vertical and horizontal dashed lines represent a $\log_2$-fold enrichment ratio of 2 and a $-\log_{10}$ *p*-value of 1.3 (*p*-value of 0.05), respectively. Significantly enriched PLP-DEs are indicated as blue dots. The data represent n = 4 biologically independent replicates. PLP-DEs = pyridoxal-phosphate-dependent enzymes, wt = wildtype.

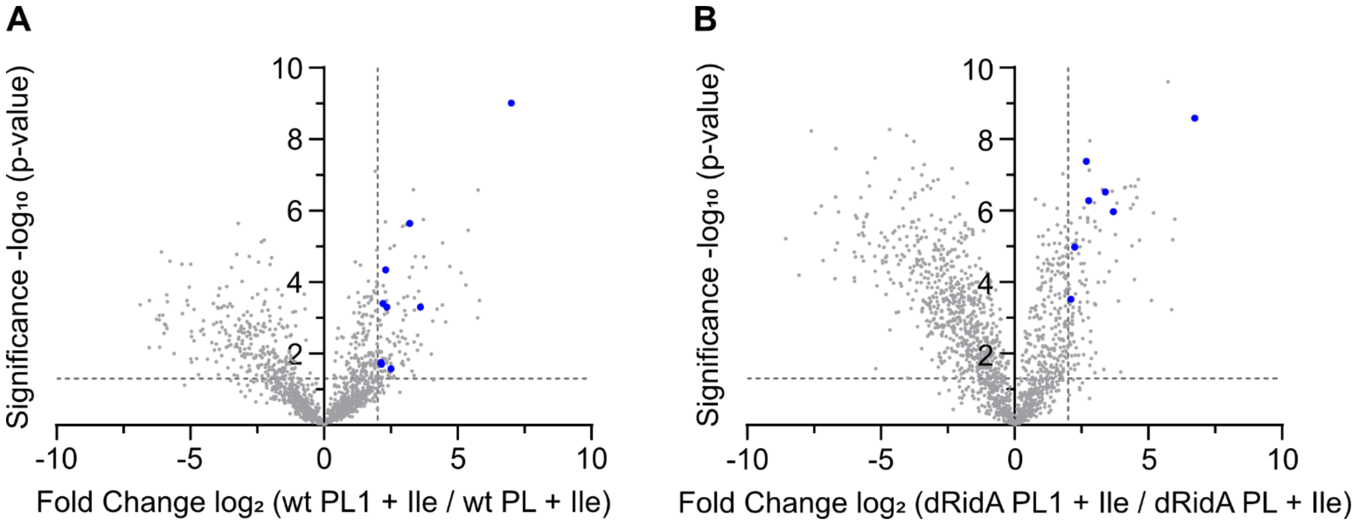

Figure S3. Profiling PLP-DEs in *S. enterica* with PL1 and Ile without $NaBH_4$ reduction (workflow #2). A and **B**, Volcano plots of *S. enterica* wildtype (wt) (A) or cells lacking RidA (dRidA) (B) grown with PL1 (1 μM) compared to those grown with PL (1 μM) and isoleucine (0.3 mM) as sole source of B6 in minimal medium. A two-sample Student's *t*-test was performed for all relevant comparisons to calculate the fold change values and statistical significance. The vertical and horizontal dashed lines represent a $\log_2$-fold enrichment ratio of 2 and a $-\log_{10}$ *p*-value of 1.3 (*p*-value of 0.05), respectively. Significantly enriched PLP-DEs are indicated as blue dots. The data represent n = 4 biologically independent replicates. PL = pyridoxal, PLP-DEs = pyridoxal phosphate-dependent enzymes, Ile = isoleucine, wt = wildtype.

A

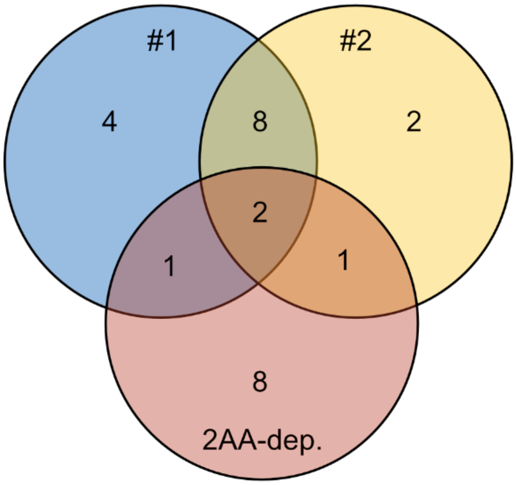

B

	Gene	#1	#2	2AA
1	*dadX*	x		x
2	*alr*	x	x	x
3	*ilvE*	x	x	
4	*bioA*	x	x	
5	*phoN*	x	x	
6	*kbl*	x		
7	*pdxK*	x	x	
8	*sufS*	x	x	
9	*selA*	x		
10	*speC*	x	x	
11	*yggS*	x		
12	*csdA*	x	x	
13	*iscS*	x	x	x
14	*arnB*	x	x	
15	*pdxY*	x		
16	*trpB*		x	
17	*ptsJ*		x	x
18	*argD*		x	
19	*ilvA*			x
20	*pdxH*			x
21	*pdxB*			x
22	*dcyD*			x
23	*tyrB*			x
24	*metB*	*		x
25	*glgP*	*		x
26	*yfdZ*	*		x

Figure S4: Venn diagram shows distribution of enriched PLP-DEs in the different experiments. A, Venn diagram representation of PLP-DEs that were enriched in each of the three experiments described in the text using probe **PL1**. **B**, Table showing the identified proteins in each workflow. The identified proteins can be determined by comparing the data in Tables S1, S2, and S3. Proteins enriched in workflow #1 are included in the blue circle, those enriched in workflow #2 are found in the yellow circle and those enriched when comparing high and low levels of 2AA are included in the red circle. PLP-DEs = pyridoxal-phosphate-dependent enzymes, 2AA = 2-aminoacrylate, * = enriched with workflow #1 with **PL2** but not **PL1**.

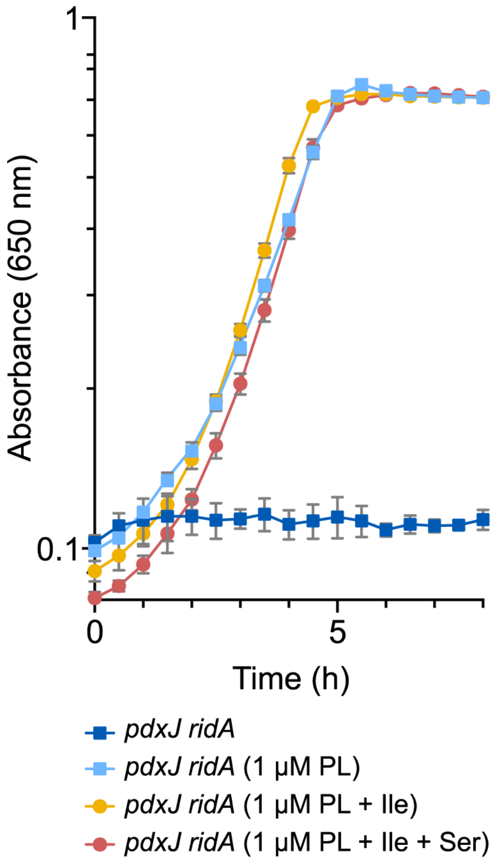

Figure S5. 2AA does not impact the salvage of PL as sole source of B6 by *S. enterica*. A mutant strain that requires exogenous B6 and lacking *ridA* was grown in minimal glucose (11 mM) medium with no addition or with PL (1 μ M) and indicated amino acid(s) in the medium. Growth was determined by monitoring optical density at 650 nm of n = 3 biological replicates over time, with the mean plotted. Strains are designated by the symbols in the included legend.

Table S1: List with PLP-DEs that were identified in the experimental approach with $NaHB_4$ reduction (workflow #1). PLP-DEs passed the $\log_2$ fold change cutoff of 2 in either one of the tested conditions. Indicated are the respective gene names, UniProt ID, fold change and *p*-values.

		**wt PL1 vs wt PL**		**wt PL2 vs wt PL**		**dRidA PL1 vs dRidA PL**		**dRidA PL2 vs dRidA PL**	
Gene name	UniProt ID	Fold change log2	-log10 (*p*-value)	Fold change log2	-log10 (*p*-value)	Fold change log2	-log10 (*p*-value)	Fold change log2	-log10 (*p*-value)
dadX	P06191	0.71	0.46	1.21	0.90	2.85	2.36	1.51	1.64
alr	P0A1A3	2.98	2.91	1.33	2.20	4.28	3.36	-1.00	1.29
ilvE	P0A1A5	1.88	1.79	0.96	2.46	2.96	3.45	1.54	2.43
bioA	P12677	2.28	3.50	0.33	1.18	2.43	2.97	0.89	2.68
phoN	P26976	3.22	4.29	-0.10	0.06	2.81	6.76	-0.90	0.95
cysM	P29848	-0.05	0.02	2.43	2.73	-0.31	0.23	2.08	3.30
kbl	P37419	2.18	4.67	-0.25	0.82	2.89	3.76	1.00	3.14
pdxK	P40192	5.47	4.32	1.21	1.18	6.58	3.96	3.73	3.26
sufS	Q7CQN5	5.06	4.52	0.55	0.61	5.02	4.25	1.70	2.68
STM4446	Q8ZK43	-	-	4.04	4.87	-	-	4.28	2.54
metB	Q8ZKN5	-0.11	0.41	0.71	1.78	-0.47	2.63	2.92	3.72
dsdA	Q8ZL08	-1.51	3.26	1.65	1.98	-0.36	0.23	2.59	2.22
STM3768	Q8ZL27	-	-	0.54	0.20	-	-	4.05	2.15
selA	Q8ZL69	3.80	5.29	1.38	1.97	3.69	3.27	2.22	2.58
glgP	Q8ZLG7	0.49	0.26	1.56	5.08	-0.29	0.20	2.60	4.82
patA	Q8ZLX7	-1.69	1.72	1.00	0.83	-2.62	2.80	2.46	3.13
speC	Q8ZM37	0.66	0.53	1.09	1.11	2.15	3.37	2.53	3.42
yggS	Q8ZM47	1.18	1.51	2.27	2.01	1.74	4.47	0.78	0.30
csdA	Q8ZMC3	3.35	5.67	1.11	1.00	3.21	4.52	1.33	1.22
iscS	Q8ZN40	0.79	0.84	0.08	0.25	2.17	5.59	1.02	4.41
yfdZ	Q8ZNA2	0.90	1.24	2.37	2.73	0.81	0.96	3.01	2.77

arnB	Q8ZNF3	3.89	3.51	0.63	1.43	3.27	2.61	0.31	0.47
pdxY	Q8ZPM8	0.64	0.74	-0.33	0.40	2.11	3.98	-0.10	0.18
bioF	Q8ZQQ7	-	-	2.06	1.87	-	-	1.13	0.47
idcC	Q8ZRN7	-0.85	1.04	1.90	1.61	-0.35	0.31	3.86	3.99

Table S2: List with PLP-DEs that were identified in the experimental approach without $NaHB_4$ reduction (workflow #2). PLP-DEs passed the log_2 fold change cutoff of 2 in either one of the tested conditions. Indicated are the respective gene names, UniProt ID, fold change and *p*-values.

		**wt PL1 vs wt PL**		**wt PL1 + Ile vs wt PL + Ile**		**dRidA PL1 vs dRidA PL**		**dRidA PL1 + Ile vs dRidA PL + Ile**	
Gene name	UniProt ID	Fold change log2	-log10 (*p*-value)	Fold change log2	-log10 (*p*-value)	Fold change log2	-log10 (*p*-value)	Fold change log2	-log10 (*p*-value)
alr	P0A1A3	3.63	5.16	2.21	3.41	4.12	6.35	2.77	6.28
ilvE	P0A1A5	1.80	3.44	3.20	5.64	2.48	5.82	3.40	6.52
trpB	P0A2K1	1.71	3.85	2.16	1.71	2.47	5.34	2.10	3.52
bioA	P12677	1.79	4.37	2.51	1.58	1.49	4.34	1.23	4.32
phoN	P26976	2.59	6.43	1.90	1.61	2.59	5.90	1.89	4.18
pdxK	P40192	6.22	5.76	7.00	9.01	6.49	6.94	6.74	8.58
ptsJ	P40193	3.98	3.54	2.14	1.76	4.10	3.52	1.99	2.21
argD	P40732	1.40	4.08	2.35	3.30	2.00	6.15	2.69	7.38
sufS	Q7CQN5	2.68	4.78	1.55	1.93	3.07	6.46	1.22	4.63
speC	Q8ZM37	1.30	3.29	2.31	4.35	1.28	5.89	2.25	4.98
csdA	Q8ZMC3	2.15	5.62	1.43	3.04	3.24	7.85	1.39	4.30
iscS	Q8ZN40	-	-	-	-	3.48	6.17	-2.64	3.71
arnB	Q8ZNF3	1.75	3.36	3.61	3.30	1.95	4.43	3.70	5.97

Table S3: List with PLP-DEs that were identified in the experimental approach under 2AA stress. PLP-DEs passed the $\log_2$ fold change cutoff of 2 (dRidA PL1 vs dRidA **PL1** + Ile) or 1 (dRidA **PL1** vs wt **PL1**) in either one of the tested conditions. Indicated are the respective gene names, UniProt ID, fold change and *p*-values.

		**dRidA PL1 vs dRidA PL1 + Ile**		**dRidA PL1 vs wt PL1**	
Gene name	UniProt ID	Fold change log2	-log10 (*p*-value)	Fold change log2	-log10 (*p*-value)
dadX	P06191	1.91	2.75	2.65	3.02
alr	P0A1A3	1.29	3.62	1.07	2.15
ilvA	P20506	1.36	3.30	1.24	1.96
ptsJ	P40193	2.55	3.25	0.56	0.63
pdxB	P60802	3.61	3.14	0.44	1.14
tyrB	P74861	0.12	0.10	1.20	2.50
metB	Q8ZKN5	-0.18	0.54	1.03	3.12
glgP	Q8ZLG7	2.82	2.71	0.87	0.63
iscS	Q8ZN40	5.88	5.48	2.95	3.42
yfdZ	Q8ZNA2	2.80	3.66	-0.03	0.03
dcyD	Q8ZNT7	2.45	3.07	0.83	2.24
pdxH	Q8ZPM9	3.14	2.16	0.24	0.26

Table S4: DIA scan windows: m/z ranges used for MS2 spectra collection.

Window	m/z Range
1	399.5-410.5
2	409.5-420.5
3	419.5-430.5
4	429.5-440.5
5	439.5-450.5
6	449.5-460.5
7	459.5-470.5
8	469.5-480.5
9	479.5-490.5
10	489.5-500.5
11	499.5-510.5
12	509.5-520.5
13	519.5-530.5
14	529.5-540.5
15	539.5-550.5
16	549.5-560.5
17	559.5-570.5
18	569.5-580.5
19	579.5-590.5
20	589.5-600.5
21	599.5-610.5
22	609.5-620.5
23	619.5-630.5

24	629.5-640.5
25	639.5-650.5
26	649.5-660.5
27	659.5-670.5
28	669.5-680.5
29	679.5-690.5
30	689.5-700.5
31	699.5-720.5
32	719.5-740.5
33	739.5-760.5
34	759.5-780.5
35	779.5-800.5
36	799.5-820.5
37	819.5-840.5
38	839.5-860.5
39	859.5-880.5
40	879.5-900.5
41	899.5-920.5
42	919.5-940.5
43	939.5-960.5
44	959.5-980.5
45	979.5-1000.5

Table S5: Parameter for MaxQuant search.

Parameter	Value
Version	2.1.0.0
Date of writing	03.01.2023 03:22
Include contaminants	True
PSM FDR	0.01
PSM FDR Crosslink	0.01
Protein FDR	0.01
Site FDR	0.01
Use Normalized Ratios For Occupancy	True
Min. peptide Length	7
Min. score for unmodified peptides	0
Min. score for modified peptides	40
Min. delta score for unmodified peptides	0
Min. delta score for modified peptides	6
Min. unique peptides	0
Min. razor peptides	1
Min. peptides	1

Use only unmodified peptides and	True
Modifications included in protein quantification	Oxidation (M);Acetyl (Protein N-term)
Peptides used for protein quantification	Razor
Discard unmodified counterpart peptides	True
Label min. ratio count	2
Use delta score	False
iBAQ	True
iBAQ log fit	True
Match between runs	True
Matching time window [min]	0.7
Match ion mobility window [indices]	0.05
Alignment time window [min]	20
Alignment ion mobility window [indices]	1
Find dependent peptides	False
Fasta file	*S. typhimurium LT2* (taxon identifier: 99287 (UP000001014), downloaded 27th of February 2023)
Decoy mode	revert
Include contaminants	True

Advanced ratios	True
Fixed andromeda index folder	
Combined folder location	
Second peptides	True
Stabilize large LFQ ratios	True
Separate LFQ in parameter groups	False
Require MS/MS for LFQ comparisons	True
Calculate peak properties	False
Main search max. combinations	200
Advanced site intensities	True
Write msScans table	False
Write msmsScans table	True
Write ms3Scans table	True
Write allPeptides table	True
Write mzRange table	True
Write DIA fragments table	False
Write DIA fragments quant table	False
Write pasefMsmsScans table	True
Write accumulatedMsmsScans table	True
Max. peptide mass [Da]	4600

Min. peptide length for unspecific search	8
Max. peptide length for unspecific search	25
Razor protein FDR	True
Disable MD5	False
Max mods in site table	3
Match unidentified features	False
Epsilon score for mutations	
Evaluate variant peptides separately	True
Variation mode	None
MS/MS tol. (FTMS)	20 ppm
Top MS/MS peaks per Da interval. (FTMS)	12
Da interval. (FTMS)	100
MS/MS deisotoping (FTMS)	True
MS/MS deisotoping tolerance (FTMS)	7
MS/MS deisotoping tolerance unit (FTMS)	ppm
MS/MS higher charges (FTMS)	True
MS/MS water loss (FTMS)	True

MS/MS water loss (FTMS for cross link)	False
MS/MS ammonia loss (FTMS)	True
MS/MS ammonia loss (FTMS for cross link)	False
MS/MS dependent losses (FTMS)	True
MS/MS recalibration (FTMS)	False
MS/MS tol. (ITMS)	0.5 Da
Top MS/MS peaks per Da interval. (ITMS)	8
Da interval. (ITMS)	100
MS/MS deisotoping (ITMS)	False
MS/MS deisotoping tolerance (ITMS)	0.15
MS/MS deisotoping tolerance unit (ITMS)	Da
MS/MS higher charges (ITMS)	True
MS/MS water loss (ITMS)	True
MS/MS water loss (ITMS for cross link)	False
MS/MS ammonia loss (ITMS)	True
MS/MS ammonia loss (ITMS for cross link)	False

MS/MS dependent losses (ITMS)	True
MS/MS recalibration (ITMS)	False
MS/MS tol. (TOF)	40 ppm
Top MS/MS peaks per Da interval. (TOF)	10
Da interval. (TOF)	100
MS/MS deisotoping (TOF)	True
MS/MS deisotoping tolerance (TOF)	0.01
MS/MS deisotoping tolerance unit (TOF)	Da
MS/MS higher charges (TOF)	True
MS/MS water loss (TOF)	True
MS/MS water loss (TOF for cross link)	False
MS/MS ammonia loss (TOF)	True
MS/MS ammonia loss (TOF for cross link)	False
MS/MS dependent losses (TOF)	True
MS/MS recalibration (TOF)	False
MS/MS tol. (Unknown)	20 ppm
Top MS/MS peaks per Da interval. (Unknown)	12

Da interval. (Unknown)	100
MS/MS deisotoping (Unknown)	True
MS/MS deisotoping tolerance (Unknown)	7
MS/MS deisotoping tolerance unit (Unknown)	ppm
MS/MS higher charges (Unknown)	True
MS/MS water loss (Unknown)	True
MS/MS water loss (Unknown for cross link)	False
MS/MS ammonia loss (Unknown)	True
MS/MS ammonia loss (Unknown for cross link)	False
MS/MS dependent losses (Unknown)	True
MS/MS recalibration (Unknown)	False
Site tables	Oxidation (M)Sites.txt
